# Re: Clinico-radiological Observations in Meconium Aspiration Syndrome - Letter to The Editor

**DOI:** 10.31729/jnma.3834

**Published:** 2018-10-31

**Authors:** Nagendra Chaudhary, Susana Lama, Shyam Kumar Mahato, Nikhil Agrawal, Santosh Pathak, Om Prakash Kurmi, Baldev Bhatia, Kailash Nath Agarwal

**Affiliations:** 1 Department of Pediatrics, Universal College of Medical Sciences, Bhairahawa, Nepal; 2 Sanjeevani Hospital & IVF Centre, Biratnagar, Nepal; 3 Vayodha Hospital Pvt. Ltd., Balkhu Chowk, Kathmandu, Nepal; 4 Department of Pediatrics, Chitwan Medical College, Bharatpur, Nepal; 5 Department of Population Health Research Institute (PHRI), McMaster University, Hamilton, Canada; 6 Centre for Population Health Research, Bhairahawa, Nepal; 7 Department of Pediatrics, Heritage Institute of Medical Sciences, Varanasi, India

**Dear Editor**,

We thank the reader for their interest in our paper and providing us an opportunity to offer our views on a few pertinent issues. The point-wise replies to the reader's query are as follows:

**Reply**:

1. MAS is defined as respiratory distress in a neonate born through meconium-stained amniotic fluid (MSAF) with characteristic radiological findings (hyperinflation and patchy opacities) whose symptoms cannot be explained otherwise.^1^ We classified meconium aspiration syndrome as a) presence of meconium stained amniotic fluid (MSAF) and staining of nails, skin and cord with meconium; b) presence of meconium below the vocal cords; c) clinical respiratory distress shortly after birth; and/or abnormal chest x-ray consistent with aspiration pneumonitis. All babies fulfilling the first and any of the remaining criteria for diagnosing MAS admitted to NICU, during the above mentioned period were included in the study.
  Presence of meconium below the vocal cords will obviously lead to respiratory distress. It is very unlikely in a neonate to have meconium below vocal cords and without respiratory distress. In the other hand, clinical presentations in MAS varies, it can range from no respiratory distress to severe distress.^2^ Therefore, the authors feel that the above used classification is as per available literature.
  The severity of MAS was graded on the basis of requirement of respiratory support as: 1) mild MAS- requiring <40% oxygen for <48 hours, 2) moderate MAS- requiring more than 40% oxygen for more than 48 hours with no air leaks, and 3) severe MAS- requiring assisted ventilation for more than 48 hours and often associated with persistent pulmonary hypertension (PPHN).^3, 4^ We also classified newborns in to severe MAS who had to be intubated and ventilated (invasive or CPAP) within 48 hours.
2. Birth asphyxia as per NNPD network is defined as- a) Moderate asphyxia: slow gasping breathing or an Apgar score of 4 to 6 at one minute, and b) Severe asphyxia: No breathing or and Apgar score of 0–3 in one minute. Although the American Academy of Pediatrics and American College of Obstetrics and gynaecology (ACOG) criteria provides more accurate classification of birth asphyxia [includes cord blood gas (metabolic or mixed acidosis, PH <7.2), Apgar score, signs of neonatal neurological dysfunction and multi-organ involvement],^5, 6^ the facility of getting cord blood gas done in majority of hospitals in low and middle income countries is still a challenge. Therefore, the use of Apgar score in such conditions can be a useful tool to assess asphyxia.
  The main aim of the study was to study the clinico-radiological observations of MAS babies. To study asphyxia, we classified newborns as no asphyxia, moderate asphyxia and severe asphyxia. We did not study the outcomes in terms of hypoxic ischemic encephalopathy (HIE) sequlae. The authors feel the newborns could have classified into no HIE, moderate HIE and severe HIE as per Sarnat and Sarnat staging. This would have helped in prognosticating the cases.
3. This study was conducted using Neonatal Resuscitation Program (NRP) 2010 in which the management of birth depends upon the vigorous/non-vigorous status of the baby.^7^
  A baby with good cry, good muscle tone and heart rate>100 is classified as vigorous. Out of the 78 MAS cases admitted, 53 (67.9%) cases were non-vigorous and in 44 (56.4%) cases, positive pressure ventilation was given but none of the cases from the vigorous group 25 (32.1 %) needed any kinds of resuscitative measures.
  The main objective of the study was to study the clinico-radiological profile of the meconium babies. Knowing the number of babies who were vigorous and non-vigorous and their relationship with the thickness of the liquor was not the objective of the paper.
4. The aim of the study was to study the clinical and radiological (chest x-ray) profiles of MAS in relation to gender, gestational age, mode of delivery, birth weight, apgar score, thickness of meconium, age at admission and the immediate outcome. We did not study the prevalence of MAS among whole deliveries conducted in the hospital or proportions of MAS among all MSL. As per our study aim, we found that the prevalence of MAS in NICU admissions is high. We found that out of 584 admitted newborns (male = 389; female = 186) during the study period, 78 (13.4%) babies had MAS, of which 56 (71.8%) were males and rest 22 (28.2%) females.
  The above comments given by the reader if addressed, could have given a clearer picture regarding MAS. This was our limitation.
5. The readers should not be misguided by the sentence that all the babies were given oxygen and IV fluids in the present study. All infants with the diagnosis of meconium aspiration syndrome (thick and thin) were managed as per neonatal resuscitation programme (NRP) guidelines. All MAS babies were admitted and monitored for respiratory distress. Those with minimal distress were waived out of oxygen as soon as possible to maintain the Spo2 of 88–95%. Feed was initiated as soon as possible. Antibiotics, inotropic support, and ventilator support was given as and when required following routine investigations for hemoglobin, total and differential leucocyte counts, platelets and C-reactive protein.
6. We apologize for the mistake in data. It was a typographical error. The mistake has been corrected.

**Table:** 

Birth Asphyxia	Thin n (%)	Thick n (%)
No	10 (41.7)	14 (58.3)
Moderate	12 (36.4)	21 (63.6)
Severe	4 (19.0)	17 (81.0)
7. There was no difference in the mean and median use. However, the data was slightly skewed to the right. The median duration of stay in NICU for thin and thick meconium babies was 4 (IQR 1–5) days and 3 (IQR 1–5) days respectively.
8. The classification of MAS as thin and thick subtypes have been used long back since 1 995.^5^ In this study a post graduate resident was well trained to identify the types of MAS (thin and thick) to nullify the variation. In this study, newborns were sub-grouped into two categories, thin and thick MSL. Thin MSL was defined as liquor having greenish yellow in color whereas liquor having dark green or tarry black or muddy in color and of thick consistency was graded as thick MSL.
  The authors agree to that further studies with new classification for MAS should be proposed with objective parameters.

## Conflict of Interest

**None.**
